# A novel CRT‐IMRT‐combined (Co‐CRIM) planning technique for peripheral lung stereotactic body radiotherapy in pinnacle treatment planning system

**DOI:** 10.1002/acm2.13461

**Published:** 2021-10-26

**Authors:** YanHua Duan, LiJun Zhou, Hao Wang, Hua Chen, HengLe Gu, Yan Shao, AiHui Feng, Ying Huang, XiaoLong Fu, Ning Jeff Yue, Kui Ma, Qing Kong, ZhiYong Xu

**Affiliations:** ^1^ Department of Radiation Oncology Shanghai Chest Hospital Shanghai Jiao Tong University Shanghai China; ^2^ Department of Radiation Oncology Fudan University Shanghai Cancer Center Shanghai China; ^3^ Department of Radiation Oncology Rutgers Cancer Institute of New Jersey Rutgers University New Jersey USA; ^4^ Clinical helpdesk Varian Medical Systems Beijing China; ^5^ Institute of Modern Physics Fudan University Shanghai China

**Keywords:** Co‐CRIM, CRT, IMRT, lung SBRT, planning strategy, VMAT

## Abstract

**Objectives:**

This study attempts to explore a novel peripheral lung stereotactic body radiotherapy (SBRT) planning technique that can balance the pros and cons of three‐dimensional conformal radiotherapy (CRT) and intensity‐modulated radiation therapy (IMRT) / volumetric modulated arc therapy (VMAT).

**Methods:**

Treatment plans were retrospectively designed based on CRT, IMRT, VMAT, and the proposed CRT‐IMRT‐combined (Co‐CRIM) techniques using Pinnacle treatment planning system (TPS) for 20 peripheral lung cancer patients. Co‐CRIM used an inverse optimization algorithm available in Pinnacle TPS. To develop a Co‐CRIM plan, the number of segments in each field was limited to one, the minimum segment area was set to the internal target volume (ITV), and the minimum monitor units (MU) of the segment was the quotient of fractional dose divided by twice the number of total fields. The performance of Co‐CRIM was then compared with other techniques.

**Results:**

For conformity index (CI), Co‐CRIM performed comparably to IMRT/VMAT but better than CRT. For gradient index (GI), Co‐CRIM was similar to IMRT/VMAT or CRT. For heterogeneity index (HI), Co‐CRIM was comparable to IMRT/VMAT, higher than CRT. The dosimetric results of spinal cord and lung with Co‐CRIM were better than CRT, comparable to IMRT, but inferior to VMAT. The MU resulted from Co‐CRIM was lower than IMRT/VMAT but higher than CRT. For plan verification γ passing rate, Co‐CRIM was higher than IMRT/VMAT, comparable to CRT. For planning time, Co‐CRIM was shorter than CRT or VMAT but similar to IMRT.

**Conclusions:**

The proposed Co‐CRIM technique on Pinnacle TPS is an effective planning technique for peripheral lung SBRT.

## 1 INTRODUCTION

As a technique that has been widely employed, stereotactic body radiotherapy (SBRT) can be used to deliver high fractional dose in few fractions, giving the tumor high‐dose precise ablation while minimizing damages to organs at risk (OARs).^1^, ^2^, ^3^ Compared with traditional radiotherapy, SBRT provides better efficacy, lower toxicity, and shorter treatment duration.^4^, ^5^, ^6^ Clinical evidence and studies have shown that the therapeutic effect of early non‐small cell lung cancer (NSCLC) patients treated with SBRT is similar to or even better than that of surgery^4^, ^5^, ^7^ and that SBRT is the major alternative therapy for patients with NSCLC who are unsuitable or unwilling to undergo surgery.^8^, ^9^, ^10^

Three‐dimensional conformal radiotherapy (CRT), along with intensity‐modulated radiation therapy (IMRT) and its advanced form volumetric modulated arc therapy (VMAT) have been widely used in SBRT. CRT may be superior to IMRT/VMAT in several aspects. In CRT, the complexity of the MLC pattern is lower. Thus, the treatment plans and deliveries are less affected by MLC uncertainties caused by software and hardware^6^ and the interaction between MLC motion and respiratory tumor movement.^11^ However, CRT is often limited in meeting the desired sparing of OARs, while providing high dose gradient conformal tumor coverage. Zhang et al.^12^ investigated that about 30% of pulmonary SBRT cases cannot be effectively treated with CRT. Not only that, CRT needs to be planned in a forward fashion, which involves constant adjustment of plan parameters to obtain the clinically acceptable treatment plan, leading to a potentially extended planning process. Moreover, the planners’ expertise plays a decisive role in the qualities of the plans, resulting in the inconsistency of plan quality. On the other hand, various studies^1^, ^13^, ^14^, ^15^ have demonstrated that IMRT/VMAT may be superior to CRT in the following aspects. IMRT/VMAT can spare OARs better while achieving high dose conformity of targets, and almost all dosimetric indexes are better than those of CRT. IMRT/VMAT uses inverse planning algorithms and offers the potentials to improve work efficiency and plan consistency. However, the IMRT/VMAT plans involve segments with small areas or low monitor units (MU), leading to poor consistency between the planned dose and the delivered dose. Additionally, the IMRT/VMAT is more susceptible to the interplay effect between MLC motion and target respiratory movement, resulting in possible compromise of target dose coverage and excessive normal tissue doses.^1^, ^6^, ^11^, ^13^, ^14^ Compared with CRT, the MUs of IMRT/VMAT are increased significantly,^14^ leading to an increase in leakage radiation.^16^

Given the pros and cons of the available radiotherapy techniques, there have been several studies on lung SBRT planning strategies. Some researchers pay more attention to factors such as dosimetry and plan design efficiency and prefer IMRT/VMAT.^13^, ^17^ Others care more about the factors such as MUs, respiratory movement, delivery accuracy, and prefer CRT.^1^, ^11^, ^14^ Most of the existing studies focused on comparing and evaluating the pros and cons of different techniques, while there is no consensus on which technique is the best strategy for lung SBRT planning.^18^ Some scholars tried to propose a hybrid planning strategy of CRT and IMRT/VMAT^6^, ^19^, ^20^, ^21^, ^22^ to combine their pros while avoiding cons. The core of these hybrid methods is to add part CRT MLC modulation to VMAT/IMRT plans which require at least two kinds of fields with different techniques or multiple optimizations. Despite all these efforts, most hybrid techniques are used for large, late‐stage lung cancer to minimize the dose to the lungs, not for small field and MLC complexity for lung SBRT. Moreover, the existing hybrid strategies suffer from a complex and long treatment planning process. Thus, it is necessary to develop a simplified hybrid lung SBRT planning strategy that integrates the advantages of dosimetry, MU, plan design time, and delivery consistency while minimizing the disadvantages of existing methods.

This study attempts to explore a novel CRT‐IMRT‐combined (Co‐CRIM) planning technique, which can be achieved through a single inverse planning optimization for peripheral lung SBRT. The proposed method aims to balance the pros and cons of CRT and IMRT/VMAT.

## 2 MATERIALS AND METHODS

### 2.1 Data collection and patient characteristics

Twenty peripheral lung cancer patients treated in Shanghai chest hospital from February 2016 to July 2018 were retrospectively enrolled in this study. All cases had early‐stage inoperable NSCLC and were consulted with at least two radiation oncologists before receiving SBRT. The prescription dose was defined depending on tumor size, location, and patient's physical condition. The detailed patient characteristics were listed in Table 1. When the study began, all the patients signed informed consent and completed their radiotherapy. The study was approved by the Institutional Ethics Committee (the committee's reference Number: KS1863).

**TABLE 1:** Patient characteristics

Factor	Value or cases
Gender	
Female	8
Male	12
Age (years)	
Mean (SD)	64 (7)
Median (range)	66 (49–76)
Clinical Stage	T_1_ N_0_ M_0_
Location of target	
Left upper lobe	4
Left lower lobe	5
Right upper lobe	4
Right middle lobe	4
Right lower lobe	3
ITV volume(cc)	
Mean (SD)	5.8 (0.49)
Median(range)	5.75 (5.07–6.86)
PTV volume(cc)	
Mean (SD)	22.26 (1.82)
Median(range)	21.79 (19.87–26.70)
Prescription dose	
50 Gy by 5 fractions	10
50 Gy by 4 fractions	10

### 2.2 Structure delineation and planning technique design

Patients were scanned with a Siemens Somatom Definition AS computed tomography (CT) Scanner System (Siemens Healthcare, Erlangen, Germany) to obtain free‐breathing CT and four‐dimensional CT (4DCT). All targets were delineated on a MIM Maestro Station (MIM Vista Corp, Cleveland, US‐OH) by experienced radiation oncologists. The gross tumor volume (GTV) was defined in the 10 phases of 4DCT, and then 10 GTVs were merged to generate internal target volume (ITV). Planning target volume (PTV) was obtained by expanding 0.5 cm of ITV in three dimensions. All structures were reviewed and approved by an independent radiation oncologist before being used for planning design. Treatment plans were planned on the averaged 4DCT using the Pinnacle treatment planning system (TPS) (V9.10, Philips Radiation Oncology Systems, Fitchburg, WI, USA) for an Edge™ linear accelerator (Varian Medical Systems, Palo Alto, CA).

Co‐CRIM used an inverse optimization process of IMRT with multiple restrictions on the number of segments, minimum segment area, and minimum MUs of each segment. In inverse planning of Pinnacle TPS, a complete field consists of many secondary fields called segments with different shapes and MUs. The above restrictions could be implemented in the Pinnacle TPS by the special setting of three fillable parameter items, namely maximum number of segments (i.e., the upper threshold for the total number of segments), minimum segment area (i.e., the lower threshold for all segment areas), and minimum segment MUs (i.e., the lower threshold for MUs of each segment). The specific operation was to set the maximum number of segments to be the same as the total field number so that there was only one segment per field, the minimum segment area to ITV, and the minimum segment MUs to the quotient of the fractional dose (cGy) divided by twice the total field number. Table 2 shows the difference between Co‐CRIM and other techniques for comparison.

**TABLE 2:** Specific settings of the proposed and other techniques

	Maximum number of segments	Minimum segment area	Minimum segment MUs
CRT	Total field number	About maximum section area of PTV	10
Co‐CRIM	Total field number	ITV volume	The fractional dose divided by twice the total field number
IMRT	10 times the total field number	2 cm^2^	10
VMAT	2° spacing of gantry angle between control points	2 cm^2^	10

Co‐CRIM employed 10 or more 6MV fields, and the angular interval of the fields was either 15 or 20 degrees. CRT and IMRT used the same field angles as Co‐CRIM, and VMAT used two arcs with the same gantry start and stop angles as other methods. All the inverse plans were planned using the Auto‐Planning (AP) module in Pinnacle TPS. The planning optimization constraints used in this study were similar to our previous research.^23^ In addition to the general constraints in Table 3, some other constraints were individually set according to the positional relationship between the target and normal tissues. The acceptance objectives of OARs were listed in Table 4.^24^, ^25^, ^26^, ^27^

**TABLE 3:** Planning constraints used for the process of inverse optimization

Structure	Objective	Constraint	Priority	Compromise
PTV	50 Gy	–	–	–
Shell_3mm_	–	*D*_max _< 28 Gy	High	Not selected
Shell_5mm_	–	*D*_max _< 25 Gy	High	Not selected
Shell_8mm_	–	*D*_max _< 20 Gy	High	Not selected
Shell_11mm_	–	*D*_max _< 17 Gy	High	Not selected
Shell_15mm_	–	*D*_max _< 14 Gy	High	Not selected
Shell_25mm_	–	*D*_max _< 9 Gy	High	Not selected
Total Lung	–	V5 < 25%	High	Selected
		V10 < 15%	High	Selected
		V20 < 6%	High	Selected

**TABLE 4:** Planning acceptance objectives for critical structures

OARs	Volume	Threshold dose (Gy)	Max dose (Gy)
Spinal cord	<0.25 cc	22.5	30
	<0.5 cc	13.5	–
Total lung	<1500 cc	12.5	–
	<1000 cc	13.5	–
	<25%	10	–
	<10%	20	–
Esophagus	<5 cc	19.5	52.5
Heart	<15 cc	32	52.5
Trachea and large bronchus	<4 cc	16.5	52.5
Great vessels	<10 cc	47	52.5
Chest wall	<30 cc	30	–

The generation of the dose limiting‐shells listed in Table 3 is briefly described as follows. First, the PTV was expanded to a specific boundary (3, 5, 8, 15 mm) to generate an intermediate structure. The intermediate structure was then subtracted from the body to generate a shell. This step was completed by pre‐written scripts. The direct machine parameter optimization (DMPO) and the collapsed cone convolution (CCC) algorithms were used for plan optimization and dose calculation.

The dosimetric coverage requirements of the target followed the recommendations of the radiation oncology working group (RTOG) 0915.^24^ Specifically, 100% prescription dose is prescribed to cover no less than 95% of the PTV volume.

It should be noted that the auto planning module (AP) in the Pinnacle TPS refers to the automation of a plan optimization. The planner still needs to add fields and optimized goals to the plan manually before the automatic process. For the peripheral lung SBRT plans in this study, the process of adding automatically optimized goals included setting a prescription dose for PTV and related dose constraints for OARs or auxiliary structures. This step was also completed by pre‐written scripts. Subsequently, the AP module was used to perform automatic optimization of the plan.

### 2.3 Evaluation index of plan quality

The dosimetric metrics of the target included the conformity index (CI), the gradient index (GI), and the heterogeneity index (HI). For OARs, the evaluated parameters included the maximum dose (Dmax) of the spinal cord; the percentage of the volume of total lung excluding ITV receiving 20 Gy (V20), 10 Gy (V10), 12.5 Gy (V12.5), and 13.5 Gy (V13.5); the mean lung dose (MLD); Dmax and the percentage of volume receiving 32 Gy (V32) of the heart; Dmax and the percentage of volume receiving 16.5 Gy (V16.5) of the trachea and large bronchus; Dmax of great vessels; and the percentage of volume receiving 30 Gy (V30) of the chest wall. In addition, the impacts of four planning techniques on the MUs per fraction, planning time, and plan verification gamma passing rate (γ) were also investigated in the study.

The CI^28^ was computed as:

(1) $$\mathrm{CI}={V_{T,\mathrm{Rx}}}^{2}/\left(V_{T}\times V_{\mathrm{Rx}}\right)$$

where *V*_T,Rx_ is the volume of target receiving a dose equal to or greater than the prescription dose, *V*_T_ is the target volume, and *V*_Rx_ is the volume receiving a dose equal to or greater than the prescription dose. The range of CI is from 0 to 1, where CI = 1 indicates that the conformability is the best, and CI = 0 indicates that there is no conformality of target dose coverage.

The GI^17^ was calculated as:

(2) $$\mathrm{GI}=V_{50\%\mathrm{Rx}}/V_{\mathrm{Rx}}$$

where *V*_50%Rx_ is the volume receiving a dose equal to or greater than half the prescription dose. A lower value of GI represents a faster dose fall‐off in normal tissue from the target.

The HI^29^ was defined as:

(3) $$\mathrm{HI}=\left(D_{2}-D_{98}\right)/D_{\mathrm{Rx}}$$

where *D*_2_ and *D*_98_ correspond to doses delivered to 2% and 98% of the PTV volume, respectively. *D*_Rx_ was the prescription dose. The lower HI value means better dose heterogeneity to the PTV.

In order to observe the impact of special settings on the consistency of the planned and delivered dose of Co‐CRIM, we conducted a study of γ index. The γ passing rate was evaluated at 2 mm/2% (5% low‐dose threshold) with the Varian onboard measuring device portal dosimetry (PD, Varian Medical Systems). Eclipse system (Varian Medical Systems, Palo Alto, CA) was then used to compare the predicted dose and measured dose.

### 2.4 Statistical analysis

Statistical analysis was performed using SPSS 22.0 (SPSS Inc., Armonk, NY). The one‐way repeated measures analysis of variance (ANOVA) was used for comparisons among different planning methods. Bonferroi test was performed for comparisons between Co‐CRIM and any other plan when the result was significant after one‐way repeated measures ANOVA. A *p*‐value less than 0.05 was considered statistically significant.

## 3 RESULTS

**FIGURE 1:**
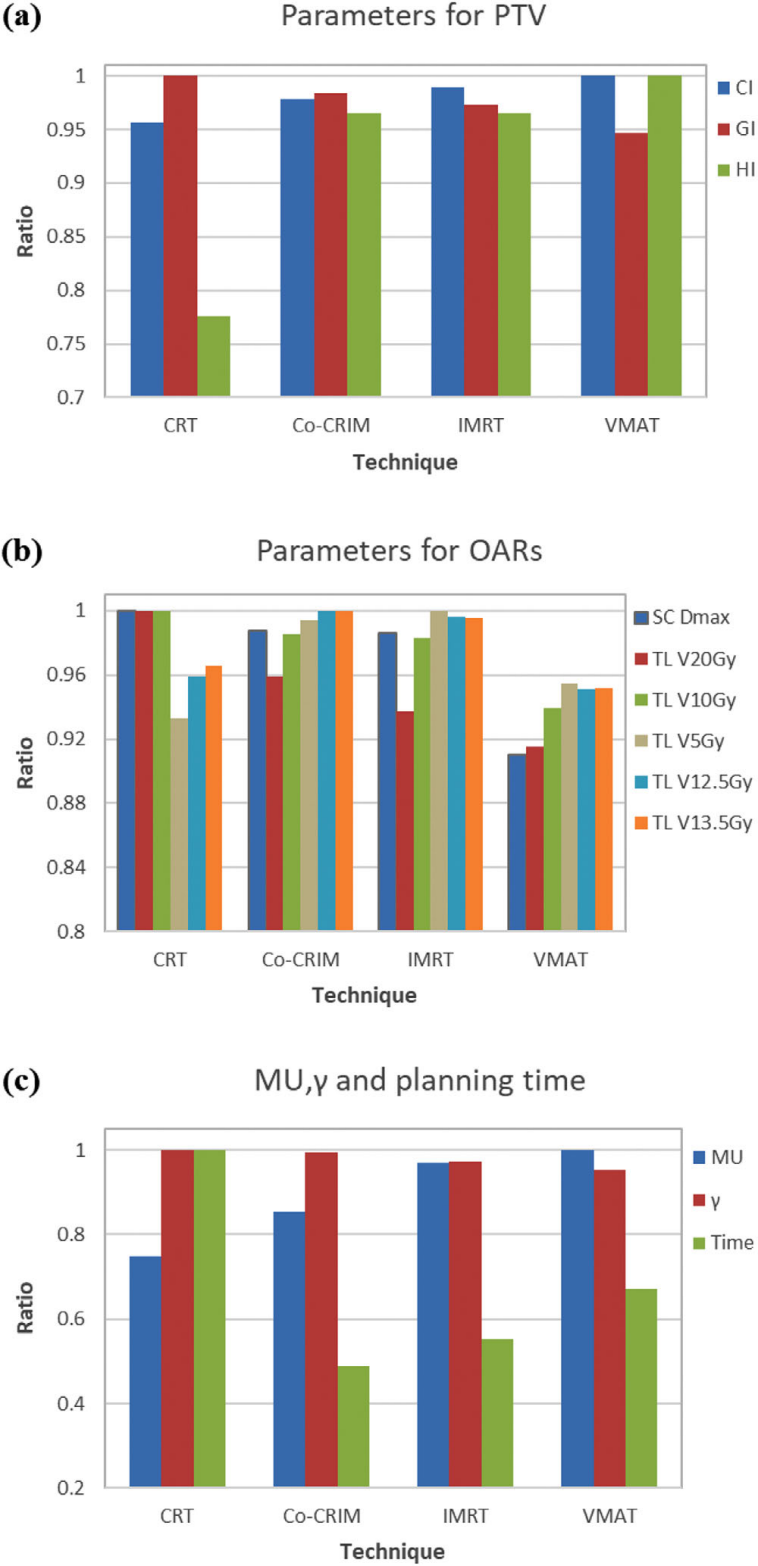
The ratios between results and maximum values for all metrics (SC, spinal cord; TL, total lung)

The dose constraints to the targets and OARs were met with the clinical requirements in all plans. Tables 5, 6, 7 present the specific numerical and statistical results for all metrics. In order to observe the pros and cons of various planning techniques clearly, Figure 1 uses a ratio to represent the metrics. The ratio is defined as the result of a parameter's value divided by the maximum value of this parameter among four techniques. Therefore, all values were normalized to 0–1 for easy display and comparison. The comparison details of the four techniques are presented in the following sections.

**TABLE 5:** Evaluations of different plans for PTV (Mean ± SD)

Parameters	CRT	Co‐CRIM	IMRT	VMAT	*p*
CI	0.87 ± 0.03	0.89 ± 0.01	0.90 ± 0.01	0.91 ± 0.02	*p* < 0.05^a^
GI	4.83 ± 0.46	4.75 ± 0.47	4.70 ± 0.51	4.57 ± 0.53	–
HI	0.45 ± 0.10	0.56 ± 0.03	0.56 ± 0.03	0.58 ± 0.07	*p* < 0.05^a^

^a^ Co‐CRIM versus CRT.

**TABLE 6:** Evaluations of different plans for OARs (Mean ± SD)

OARs	Parameters	CRT	CO‐CRIM	IMRT	VMAT	P
Spinal cord	D_max_ (Gy)	7.03 ± 1.84	6.94 ± 1.69	6.93 ± 1.71	6.40 ± 1.40	*p* < 0.05^*^, ^b^
Total lung	V20 (%)	3.20 ± 0.85	3.07 ± 0.83	3.06 ± 0.82	2.93 ± 0.81	*p* < 0.05^*^, ^b^
	V10 (%)	7.59 ± 1.53	7.48 ± 1.71	7.46 ± 1.68	7.13 ± 1.60	*p* < 0.05^*^, ^b^
	V5(%)	0.13 ± 0.03	0.13 ± 0.03	0.13 ± 0.03	0.13 ± 0.03	*p* < 0.05^*^, ^b^
	V12.5(cc)	195.71 ± 64.84	204.07 ± 69.05	203.28 ± 68.32	194.12 ± 63.84	*p* < 0.05^*^, ^b^
	V13.5(cc)	179.30 ± 61.28	185.73 ± 64.55	184.96 ± 63.46	176.82 ± 59.58	*p* < 0.05^*^, ^b^
	MLD(Gy)	2.81 ± 0.52	2.76 ± 0.52	2.76 ± 0.52	2.69 ± 0.50	*p* < 0.05^*^, ^b^
Esophagus	Dmax	9.42 ± 2.61	8.61 ± 2.34	8.59 ± 2.39	8.26 ± 2.03	–
Heart	V32Gy(cc)	0.04 ± 0.19	0.04 ± 0.16	0.07 ± 0.30	0.04 ± 0.16	–
	Dmax	15.76 ± 11.43	15.38 ± 10.95	15.32 ± 11.58	15.35 ± 11.41	–
Trachea and large bronchus	V16.5 Gy(cc)	0.11 ± 0.30	0.02 ± 0.06	0.01 ± 0.04	0.02 ± 0.07	–
	Dmax	10.90 ± 7.24	10.22 ± 6.75	9.68 ± 6.38	9.62 ± 6.58	–
Great vessels	Dmax	17.22 ± 9.49	16.21 ± 8.72	16.13 ± 9.03	16.10 ± 9.19	–
Chest wall	V30Gy(cc)	11.60 ± 8.83	10.77 ± 8.29	10.45 ± 8.12	11.05 ± 8.27	–

* CO‐CRIM versus CRT.

^b^ CO‐CRIM versus VMAT.

**TABLE 7:** Evaluations of different plans for MU, γ, and planning time

Criteria	CRT	Co‐CRIM	IMRT	VMAT	*P*
MU/F	1840 ± 298	2103 ± 334	2387 ± 463	2461 ± 512	*p* < 0.05^a^, ^b^, ^c^
γ (%)	98.44 ± 1.72	97.88 ± 1.30	95.78 ± 1.99	93.84 ± 2.45	*p* < 0.05^b^, ^c^
Time(mins)	48.23 ± 6.55	23.55 ± 3.33	26.68 ± 3.01	32.35 ± 4.28	*p* < 0.05^a^, ^b^

^a^ Co‐CRIM versus CRT.

^b^ Co‐CRIM versus VMAT.

^c^ Co‐CRIM versus IMRT.

### 3.1 Case example

**FIGURE 2:**
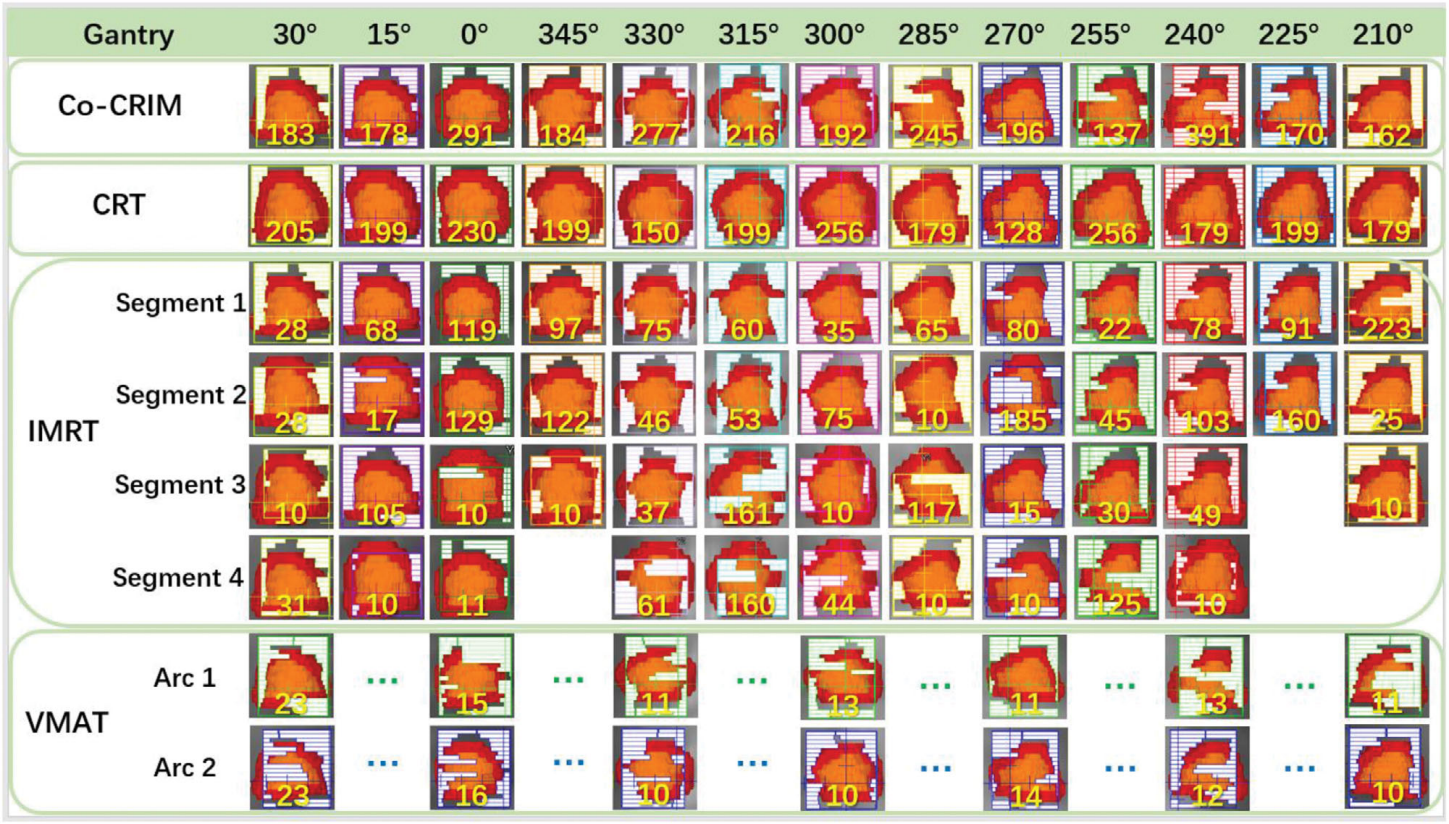
Field details of the four techniques (red area: PTV, orange area: ITV, yellow number: the number of MU)

**FIGURE 3:**
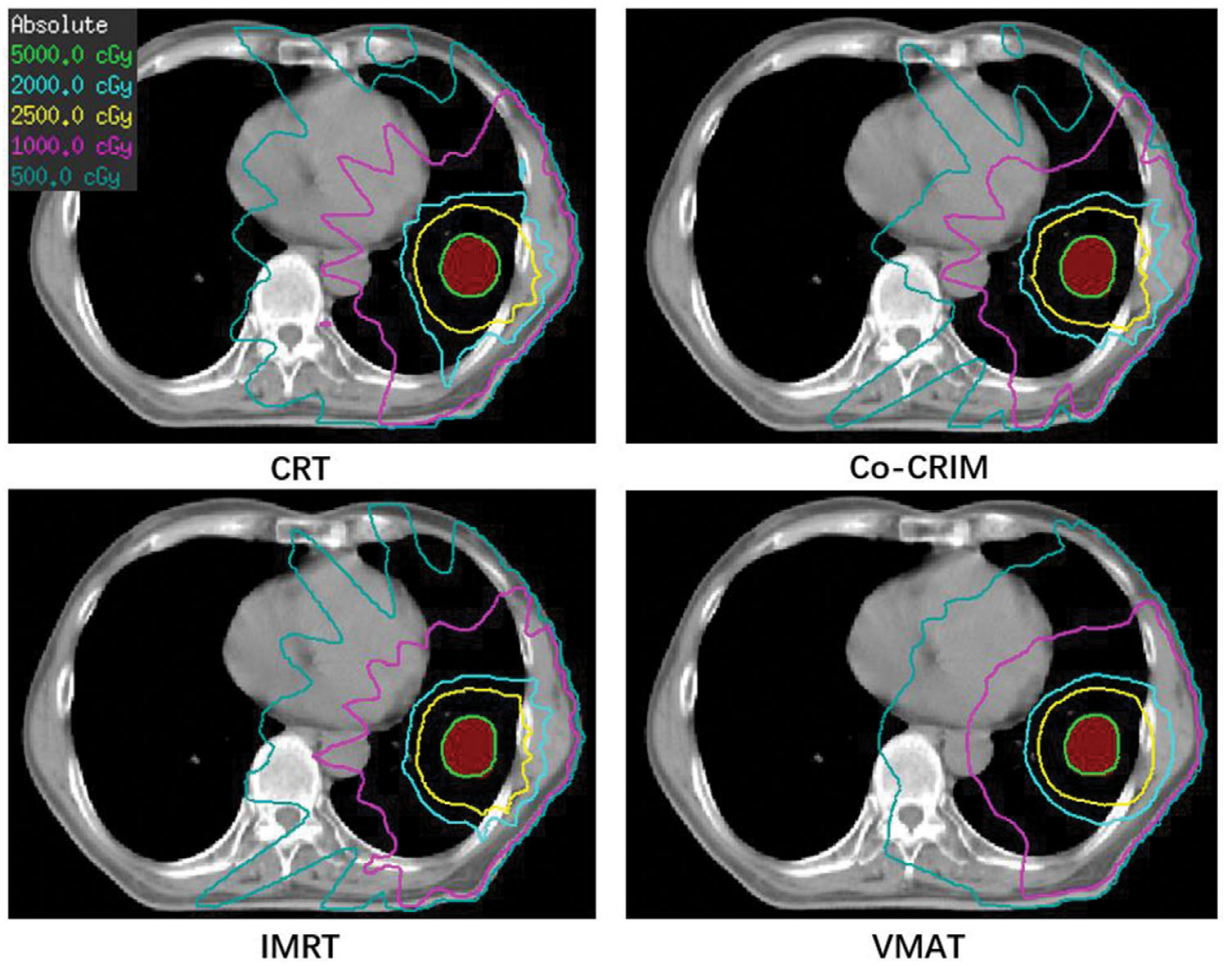
An example of the dose distribution of different techniques (red‐shaded area: PTV)

**FIGURE 4:**
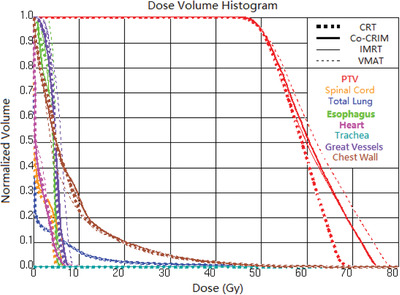
An example of the DVH of different techniques (red‐shaded area: PTV)

Figures 2, 3, 4 show an example of the field details, dose distributions, and dose‐volume histogram (DVH) for the four techniques of a same patient, respectively. The prescription dose of this patient was 50 Gy (fractional dose 12.5 Gy) and the ITV was 5.78 cc. The maximum number of segments, minimum segment area, and minimum segment MUs of the Co‐CRIM plan were set to 13 (number of total fields), 5.78 cm^2^ (the ITV), and 48 (1250/(13*2)), respectively. Figure 2 shows that IMRT had more segments, some of which were as low as 10MU, and VMAT also had smaller segments and lower MU for each segment compared to Co‐CRIM. Figure 3 shows the isodose of prescription (50 Gy), 50% prescription dose (25 Gy), 20 Gy, 10 Gy, and 5 Gy for four techniques, and only the CRT had a visually observable 50 Gy isodose that did not conform PTV. In comparison, there was no obvious difference in the 25 Gy isodose for all techniques. VMAT and CRT had the smallest and largest areas of 25 Gy isodose, respectively, and that of Co‐CRIM was between them. It can be seen from Figure 4 that VMAT and CRT had the highest and lowest hotspots, respectively, while Co‐CRIM and IMRT were in the middle and similar.

### 3.2 Evaluations of PTV in different plans

Table 5 lists the dosimetric parameter comparisons of all the techniques for PTV. For CI, Co‐CRIM (0.89) was comparable to IMRT/VMAT (0.90/0.91) (*p* = 0.06/0.05), but significantly better than CRT (0.87) (*p* = 0.04). However, the statistical differences were not found after one‐way repeated measures ANOVA in GI among different techniques, indicating that Co‐CRIM (4.75) obtained the GI comparable to CRT/IMRT/VMAT (4.83/4.70/4.57). For HI, Co‐CRIM (0.56) was similar to IMRT/VMAT (0.56/0.58) (*p* > 0.05), but significantly higher than CRT (0.45) (*p* = 0.006). In addition, hotspots (defined as the dose greater than 150% prescription dose in this study) outside of ITV were found in 5 out of 20 CRT plans but not in other techniques.

### 3.3 Evaluations of OARs in different plans

Table 6 lists the dosimetric parameter comparisons for OARs. Dmax of the spinal cord, V20, V10, V5, V12.5, V13.5 of the total lung, and MLD all showed a trend of gradual improvement (decrease) in the order of CRT, Co‐CRIM, IMRT, and VMAT. Although the numerical difference was small, statistical differences were found in part results. The dosimetric results of the spinal cord and total lung from Co‐CRIM were significantly lower than those of CRT (*p* <0.05), comparably to those of IMRT (*p* > 0.05), but higher than those of VMAT (*p* < 0.05). No statistical differences between Co‐CRIM and other techniques were found in other OARs metrics (*p* > 0.05).

### 3.4 Evaluations of MU, γ passing rate, and planning time in different plans

Table 7 tabulates the mean fractional MUs, γ, and planning time of different techniques. For MU, Co‐CRIM (2103) was significantly lower than IMRT/VMAT (2387/2461) (*p* = 0.002/0.001), but higher than CRT (1840) (*p* = 0.002). For γ, Co‐CRIM (97.88%) was significantly higher than IMRT/VMAT (95.78%/93.84%) (*p* = 0.046/0.037), and comparable to CRT (98.44%) (*p* = 1.000). For planning time, Co‐CRIM (23.55 min) was significantly shorter than CRT (48.23 min) or VMAT (32.35 min) (*p* < 0.001), which could also be observed obviously in Figure 1. However, no statistical difference was found between Co‐CRIM and IMRT (26.68 min) (*p* = 0.054).

## 4 DISCUSSIONS

SBRT has become one of standard treatments for early stage NSCLC patients who cannot be operated on.^6^ With the development of radiotherapy technique, the planning and delivery techniques of SBRT have advanced from CRT to IMRT/VMAT. Both CRT and IMRT/VMAT techniques have their own pros and cons. In general, CRT can provide a good consistency between planned and delivered dose. However, a long planning time is required to achieve desired dose distributions.^11^ IMRT/VMAT are better than CRT regarding target and normal tissue dosimetry,^1^, ^13^, ^14^, ^15^ but their delivery accuracy is relatively poor. In addition, the MUs of IMRT/VMAT are generally much higher than CRT.^14^ It is still not clear which technique is the best planning technique for lung SBRT.

In this study, a Co‐CRIM planning strategy was proposed to incorporate the advantages of both CRT and IMRT/VMAT while avoiding their respective disadvantages partly. Specifically, Co‐CRIM can retain the dosimetric advantages of IMRT/VMAT while effectively reducing MUs and increasing the γ pass‐rate. Moreover, Co‐CRIM can retain the high delivery accuracy of CRT while shortening the planning time.

This study only focused on the difference between the proposed technique and other techniques, so the statistical significance of any other two techniques for comparison was not shown in the results section.

The comparison results of Co‐CRIM to CRT, IMRT, and VMAT were consistent with those in previous studies.^13^, ^14^, ^15^, ^17^, ^30^, ^31^ For the results for PTV in Table 5, Co‐CRIM could obtain conformity as good as IMRT/VMAT and outperformed CRT (lower CI). The comparable GI of the four techniques indicated a similar dose fall‐off outside the target. Co‐CRIM and IMRT/VMAT performed similar dose uniformity (HI), while CRT provided a more uniform dose (lower HI) than other methods. As we all know, HI is a parameter reflecting dose uniformity closely related to hotspots. In clinical practice, the location of hotspots is also an important factor to be considered. Although CRT could obtain better uniformity in this study, hotspots outside ITV were found in 25% of the CRT plans. For fractionated cases, dose uniformity may be critical as intra‐ and interfraction uncertainties or shifts could cause serious adverse effects when hotspots occur near sensitive regions.^32^ Therefore, CRT should be cautiously chosen for patients with critical organs adjacent to the target. By contrast, Co‐CRIM could restrict the hotspots into ITV, showing that a significant advantage of inverse planning is to allow the hotspot position in the center of the ITV rather than at the periphery. On the other hand, with the same peripheral dose, a higher dose of ITV for SBRT may translate to enhanced clinical efficacy in treating hypoxic tumors. Therefore, high HI is not a disadvantage of a SBRT plan on the premise that the hotspots are inside ITV.^32^ In summary, as far as PTV was concerned, Co‐CRIM could well retain the dosimetric advantages of IMRT/VMAT and outperform CRT.

From the results for OARs listed in Table 6, although Co‐CRIM performed not as well as VMAT in terms of the spinal cord and total lung, the numerical differences were very low. Actually, the maximum absolute differences between the two techniques were just 0.54 Gy (1.08% of the prescription dose) for the spinal cord. The V20, V10, V5, V12.5, V13.5, and MLD of the total lung obtained by Co‐CRIM were lowered by 4.06%, 1.45%, 5.97%, 4.10%, 3.46%, and 1.78%, respectively, compared with CRT, which would potentially reduce the incidence of radiation pneumonia. In addition, the reason why we did not find differences in esophagus, heart, trachea and large bronchus, and great vessels may be that all enrolled cases were patients with peripheral lung cancer whose targets were far away from above tissues, resulting in irregularly low doses of them. In addition, although the chest wall is an organ close to peripheral tumor, we found no statistical difference in the V30 Gy (cc) of the chest wall in different techniques. The reason may be that the chest wall is a thin and long structure, which causes uncertainty in the 30 Gy coverage of it. Note, since the lung was an important OAR in this study, as many metrics as possible were used for evaluation. In addition to the metrics recommended by American Association of Physicists in Medicine (AAPM) and RTOG (V20, V12.5, V13.5), V10, V5, and MLD were also employed. Besides, the dose volumes of part OARs were not listed if their values were 0. For example, this study did not list the results of V19.5 for esophagus recommended by AAPM and RTOG because the Dmax of the esophagus of all cases was lower than 19.5 Gy. In short, for OARs, although Co‐CRIM still could not compete with VMAT in part OARs sparing, it improved the normal tissue doses over CRT and could perform similarly to IMRT.

The data presented in Table 7 indicate that although Co‐CRIM still could not compete with CRT in terms of MU, it could effectively improve the shortcomings of high MUs from IMRT/VMAT. Moreover, as shown in Figure 2, the segments of IMRT and VMAT had smaller areas and lower MUs when compared with Co‐CRIM, which would increase the uncertainty of dose delivery. Note, since this was a retrospective study, the actual beam delivery time could not be counted. When the delivery dose rate was the same, the difference in beam‐on time would be the same as that of MU in this study. CRT, IMRT, and VMAT had gradually complicated fields and segments, which result in gradually decreasing γ. The results of γ indicated that Co‐CRIM could effectively improve the consistency of the TPS calculated and delivered doses of IMRT/VMAT, and achieve a plan verification γ passing rate similar to CRT. Also, Co‐CRIM could reduce the long planning time of CRT. The long planning time of VMAT may be attributed to the prolonged automatic optimization process caused by the complexity of the VMAT plans.

To sum up, Co‐CRIM can be regarded as an inverse conformal technique based on IMRT with special optimized parameters. Since the IMRT technique is widely used in SBRT plans, the proposed method is theoretically reasonable. However, Co‐CRIM is not as good as VMAT in terms of normal tissue sparing. Therefore, VMAT should be used when the OAR is difficult to reach a safe dose to minimize normal tissue damage. In addition, the MU of the Co‐CRIM technique is higher than that of CRT, which indicates a longer beam‐on time. Therefore, the CRT plan may be more suitable for patients with poor physical conditions or involuntary movement to reduce the uncertainty of delivery dose.

In conventional IMRT plan of SBRT, 10 or more segments are usually included (see the example in Figure 2). In this particular study, we set the maximum number of segments as the total number of beams so that each beam contains only one segment. It is expected that this can reduce the modulation and thus reduce the number of MU. The results are as expected (see Table 7 for details). Also, the minimum segment area is an important parameter and set based on experiences of our institution and other sister institutions. We set the minimum segment area to ITV volume. Theoretically, the minimum segment area should be set to be approximately but slightly smaller than the projection area of the PTV so that the proposed technique can realize the advantages of the CRT effectively. However, the Pinnacle TPS cannot calculate the projection area. In clinical practice, the conformity of the target will become poor if the minimum segment area is too large, while the delivery accuracy of the plan will decrease if the setting is too small. In order to achieve accurate dose delivery, the irradiation area of all segments should cover ITV. After repeated experiments, we found that when the minimum segment area is set to the ITV, almost all segments can cover ITV (as shown in Figure 2). Moreover, Pinnacle TPS can calculate the volume of any structure quickly and easily, which provides convenience for the planning process. While the minimum segment area is not constrained, smaller and more irregular segments will be formed (see Figure 2), which will affect the plan verification passing rate. The final results are consistent with the theory (as the results in Table 7). In addition, the minimum segment MUs was set to be the quotient of the fractional dose (Rx/F) (cGy) divided by twice the total field number. In general, the average MU of each beam was more than Rx/beam number. This study set the minimum segment MU as Rx/(2*beam number). The reason is that each beam in a plan has different weight. The proposed setting for minimum segment MU will ensure that the MUs in each beam are not too low to keep a high delivery accuracy. At the same time, the suboptimal weight distribution due to high segment MUs will be avoided in the process of inverse‐planning optimization.

It should be noted that all the cases selected for this study were peripheral lung cancer patients. Whether the conclusion drawn from this study is applicable to the auto‐planning process for targets located in other lung regions needs to be further evaluated and confirmed. In future studies, more types of lung cancer tumors need to be included for classification research to determine the application scope of the proposed technique. It should also be pointed out that this study was conducted using the auto‐planning module of Pinnacle TPS. The conclusion may be different if other modules or other TPS systems are used. A similar technique can also be used for other TPS, but the impact of different planning techniques and the specific parameter settings during the planning process on other planning systems must be further studied using methods similar to this study or other measures based on specific system characteristics. Besides, this study did not involve anatomical and geometric features of the target; thus, whether the shape of the tumor (i.e., spherical vs. irregularly shaped), the size of the tumor, or other anatomical features affect the results from the proposed technique is not determined. A study found that the difference in the dose fall‐off outside the target between the 6MV IMRT and VMAT SBRT plans of the same patient had little dependence on the geometric characteristics of the target.^33^ The reason maybe that SBRT has a smaller target volume and higher dose conformability than conventional radiotherapy, so that the dose is less dependent on the target shape. Moreover, although the anatomical factors may impact the plan results of different patients, it affects little on the relative difference of the results obtained by the same patient using different techniques in this study. While our conclusion was just derived from current data, the influence of geometric and anatomical factors on other evaluation parameters of different techniques still needs to be explored with a larger patient cohort, and the evidence can be used to prove whether the technique can be generalized to all cases or it is only applicable under certain circumstances.

## 5 CONCLUSION

The proposed Co‐CRIM method is an effective peripheral lung SBRT inverse‐planning technique using Pinnacle TPS to incorporate the pros of CRT and IMRT/VMAT while partly avoiding their cons. The Co‐CRIM obtains dosimetric results similar to IMRT and better than CRT, and it can get improved MU and delivery accuracy compared to IMRT/VMAT. Moreover, it can achieve a shorter planning time than CRT and VMAT. Therefore, the Co‐CRIM technique based on Pinnacle TPS can be a possible clinical planning option for peripheral lung SBRT.

## CONFLICT OF INTEREST

None.

## FINANCIAL SUPPORT

Nurture projects for basic research of Shanghai Chest Hospital (No. 2019YNJCM05).

## AUTHOR CONTRIBUTIONS

YanHua Duan and LiJun Zhou were involved in conceptualization, investigation, methodology, software, data curation, and writing original draft preparation, and review/editing; Hao Wang was involved in conceptualization, methodology, and software; Hua Chen, HengLe Gu, Yan Shao, and Ning Jeff Yue were involved in writing review and editing; AiHui Feng and Ying Huang were involved in data curation, visualization, and writing review and editing; Kui Ma was involved in technical support of software and hardware; ZhiYong Xu, Qing Kong, and XiaoLong Fu were involved in conceptualization, methodology, project administration, supervision, and writing original draft preparation and review/editing.

## Data Availability

The data that support the findings of this study are available from the corresponding author upon reasonable request.
